# A general modular framework for gene set enrichment analysis

**DOI:** 10.1186/1471-2105-10-47

**Published:** 2009-02-03

**Authors:** Marit Ackermann, Korbinian Strimmer

**Affiliations:** 1 Biotechnology Center, Technical University Dresden, 01062 Dresden, Germany; 2 Institute for Medical Informatics, Statistics and Epidemiology, University of Leipzig, Härtelstr, 16-18, 04107 Leipzig, Germany

## Abstract

**Background:**

Analysis of microarray and other high-throughput data on the basis of gene sets, rather than individual genes, is becoming more important in genomic studies. Correspondingly, a large number of statistical approaches for detecting gene set enrichment have been proposed, but both the interrelations and the relative performance of the various methods are still very much unclear.

**Results:**

We conduct an extensive survey of statistical approaches for gene set analysis and identify a common modular structure underlying most published methods. Based on this finding we propose a general framework for detecting gene set enrichment. This framework provides a meta-theory of gene set analysis that not only helps to gain a better understanding of the relative merits of each embedded approach but also facilitates a principled comparison and offers insights into the relative interplay of the methods.

**Conclusion:**

We use this framework to conduct a computer simulation comparing 261 different variants of gene set enrichment procedures and to analyze two experimental data sets. Based on the results we offer recommendations for best practices regarding the choice of effective procedures for gene set enrichment analysis.

## Background

The analysis of "enrichment" of gene sets is a natural extension of the study of differential expression of individual genes. Focusing on sets of genes rather than on individual genes has several benefits. From a statistical point of view the analysis of groups instead of individual genes is advantageous as this typically increases power and reduces the dimensionality of the underlying statistical problem. From the biological perspective gene set enrichment analysis allows one to ask (and answer!) questions that are of direct interest to the understanding of the functional mechanism in a cell: is a certain pathway activated in a given tissue under some treatment X? Is the pathway more active than other pathways? These questions directly relate to various null models for gene sets.

Therefore, for good reason a substantial number of statistical procedures to assess gene set enrichment have been introduced in the last few years – see Table 1 and references [1-36] for an overview. Given this extensive literature, biologists are now confronted with the difficult choice of a gene set method that is best suited to analyze their data at hand. So far, a "taxonomy" of enrichment analysis approaches and a systematic comparison is lacking.

**Table 1:** Overview over statistical algorithms for the analysis of gene set enrichment.

**Method**	**References**
**overrepresentation analysis**	Draghici *et al. *[1], Hosack *et al. *[2], Zhang *et al. *[3], Kathri and Draghici [4], Vêncio and Shmulevich [6]
**gene set enrichment analysis**	Mootha *et al. *[7], Subramanian *et al. *[8], Barry *et al. *[9], Zahn *et al. *[10], Efron and Tibshirani [11], Keller *et al. *[12]
**average of single gene statistics**	Pavlidis *et al. *[13], Tian *et al. *[14], Smyth [15], Jiang and Gentleman [16], Gentleman [17]
**parametric methods**	Kim and Volsky [18], Dinu *et al. *[19]
**random-set methods**	Newton *et al. *[20]
**FDR-based method**	Efron [21]
**globaltest**	Goeman *et al. *[22]
**GlobalAncova**	Mansmann und Meister [23], Hummel *et al. *[24]
**Hotelling's ***T*^2^**-test**	Kong *et al. *[25], Dinu *et al. *[19]
**further procedures**	Rahnenführer *et al. *[26], Edelman *et al. *[27], Lewin *et al. *[28], Nacu *et al. *[29], Adewale *et al. *[30], Läuter *et al. *[31]
**reviews**	Goeman and Bühlmann [5], Liu *et al. *[32], Chen *et al. *[33], Nam and Kim [34], Song and Black [35], Dopazo [36]

**Figure 1:**
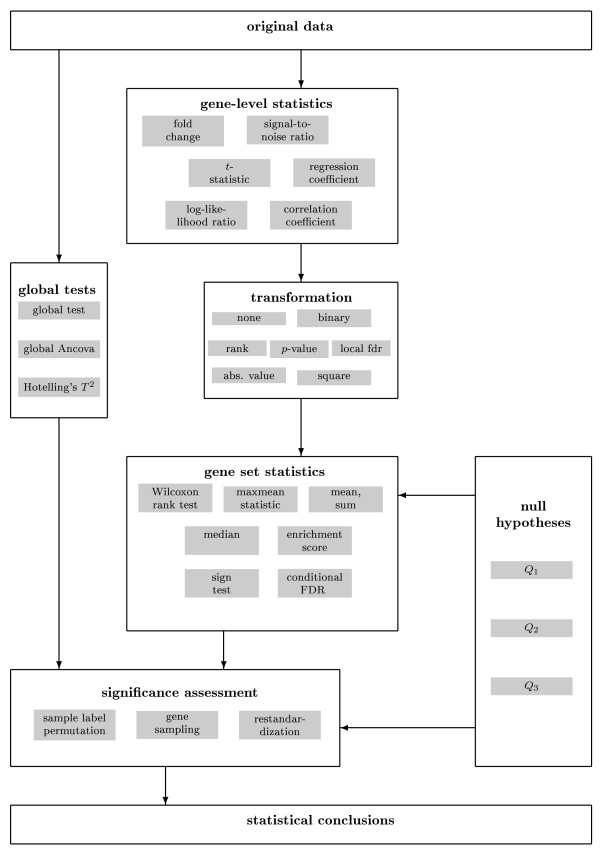
**Schematic overview of the modular structure underlying procedures for gene set enrichment analysis**.

The objective of the present work is three-fold. First, we present a meta-theory of gene set enrichment analysis in the form of a general modular framework (Fig. 1). This modular scheme encompasses most if not all published approaches for gene set analysis, and hence allows to study the interplay among the various methods. Second, based on this framework we conduct a principled comparison investigating 261 variants of gene set enrichment procedures. From extensive computer simulations and the analysis of two experimental data sets, we offer specific recommendations for conducting an effective gene set analysis. Third, we present an extensive survey of existing statistical methods for detecting enriched gene sets. This is found in the *Appendix *and forms the basis of the proposed modular framework.

## Methods

In this section we describe the key result from our studies: a modular framework that provides a taxonomy for gene set analysis. In the *Appendix *we have compiled a very comprehensive overview of the respective approaches that underlie our proposed meta-theory of gene set enrichment.

### Modular structure underlying procedures for gene set enrichment analysis

Our suggestion for a common modular framework for gene set enrichment analysis methods is depicted in Fig. 1. The scheme consists of five distinct modules: the calculation of a gene-level statistic, an optional transformation of these statistics, the choice of a null hypothesis, the computation of a gene set statistic and the significance assessment. The first two modules correspond to an analysis of differential expression on the individual gene level. The remaining three modules process these results further to obtain an assessment of gene set enrichment. A separate track in this diagram is provided for a module for conducting global tests.

We will now describe each module in more detail. Note that the scheme presented in Fig. 1 is both descriptive as well as generative: by selecting a method in each box one arrives at some specific approach for detecting gene set enrichment. To our knowledge, all enrichment approaches published so far fit into this scheme. In addition, there are also many combinations of modules that will lead to a "novel" method (i.e. one that has not yet been explicitly described in the literature). However, note that some modules are tightly interconnected, e.g., the choice of null hypothesis and of significance assessment. These dependencies need to be kept in mind when setting up a gene set analysis.

### Description of individual modules

#### Gene-level statistics

In all the univariate approaches (cf. *Appendix*), the first step in conducting a gene set enrichment analysis is to assess the amount of differential expression of the individual genes. Choosing a suitable test statistic for this purpose has been discussed extensively in the literature, for an overview we refer, e.g., to [37] and references therein.

Typically, the gene-level statistic will be selected from the following list:

• fold change,

• signal-to-noise ratio,

• (regularized) *t*-statistics,

• (shrinkage) correlation coefficient,

• coefficient of ANOVA or linear/logistic regression, and

• (penalized) log-likelihood ratio.

Due to the small sample size found in genomic data in most instances the regularized versions of these test statistics (via penalization of likelihood, suitable Bayes priors, Stein shrinkage, etc.) are preferable.

#### Transformation of gene-level statistics

The second step of univariate procedures for detecting gene set enrichment consists of the transformation of the local gene statistic. Suitable options suggested in the literature include:

• no transformation,

• absolute values,

• squared values,

• binary transformation,

• ranks,

• *p*-values or Bayesian posterior probabilities, and

• (local) false discovery rates.

There are many instances when a transformation is beneficial, for example in order to improve robustness, or to account for both up- and down-regulation. The binary transformation is an extreme case of ranking considering only two classes (e.g., "differentially expressed" versus "non-differentially expressed"). Many of the adhoc procedures used by biologists to declare differential expression (such as based on fold change and some kind of arbitrary rules) fall into this category. Also note that binary transformation combined with testing gene set membership is equivalent to the contingency table approach (cf. *Appendix*).

The influence of the transformation of the gene-level statistics on the results of gene set enrichment procedures is shown by Newton *et al. *[20]. These authors compare the power properties of averaging and selection test statistics, i.e. methods based on the mean of local statistics like the *t*-statistic and methods where the mean over the binary indicator of differential expression is used. Newton *et al. *[20] find that selection methods work better when the gene set enrichment is characterized through a small number of highly differentially expressed genes, whereas averaging is able to detect enriched sets consisting of a large number of genes with subtle increase or decrease of the expression levels.

#### Null hypotheses

A factor that strongly influences the general setup of an enrichment procedure is the choice of the null hypothesis. The two most common choices are termed *Q*_1 _and *Q*_2 _[14].

*Q*_1 _is called the "competitive null hypothesis" and corresponds to the case where the association between the genes in the set and the phenotype is compared with the association of the remainder of the genes and the phenotype. Note that this implies a model where the genes are the sampling units and the association between the samples and the phenotypes is fixed. Thus, testing this hypothesis is conditional on the gene-level scores. The "complete null hypothesis" [16] that all the gene sets under consideration have the same differential expression can be seen as a special case of *Q*_1_.

In contrast, *Q*_2 _is the "self-contained null hypothesis" that focuses on the given gene set without considering genes outside of this group. It compares the association of the gene set and the phenotype with that of random phenotypes. Under *Q*_2 _the sampling units are the phenotypes while the gene set membership is fixed. A special case of the self-contained null hypothesis is the "global hypothesis" that there are no differentially expressed genes at all.

A third possible null model, here called *Q*_3_, is the "nested null hypothesis", where differential expression of the genes in the gene set is compared to the differential expression of all genes under consideration (both inside and outside the gene set). This model is motivated by the FDR-based approach described in Efron [21], where FDR and conditional FDR values are compared.

For understanding gene set enrichment procedures it is absolutely essential to understand the implications of the various null models [5]. For instance, if there are many differentially expressed genes in all gene sets, testing under the self-contained model *Q*_2 _is quite likely to indicate enrichment, even though the particular gene set under investigation might not show any special "enrichment" compared to other gene sets.

Furthermore, as Goeman and Bühlmann [5] point out, the aim of a gene expression experiment is to assess whether the transcript levels of the genes differ between the different phenotypes. Thus, it is based on a model where the phenotypes are the sampling units. Consequently, one would repeat the experiment with different samples and not with different genes to verify the results. In contrast, the competitive hypothesis *Q*_1 _assumes that the genes are the sampling units, turning the experimental design upside down. Therefore, some authors [38,33] consider *Q*_1 _only to be "of limited utility" for statistical analysis.

#### Gene set statistics

A further step in an enrichment analysis is the computation of a gene set statistic. Examples include:

• the sum or the mean of the (transformed) single gene statistics,

• the median of the (transformed) single gene statistics,

• the (modified) Kolmogorov-Smirnov statistic,

• the maxmean statistic,

• the Wilcoxon rank sum test statistic,

• the sign test statistic, and

• the conditional local FDR.

The question of which gene set statistic is optimal in a given setting is subject to ongoing discussion. For example, Efron and Tibshirani [11] show that their maxmean statistic is more powerful than a Kolmogorov-Smirnov test or an average of local statistics. Jiang and Gentleman [16] call attention to the problem of robustness against outliers and advocate summaries such as the median or the sign test statistic.

#### Significance assessment

The last step of both the univariate and the global and multivariate procedures (cf. *Appendix*) for enrichment analysis is the assessment of significance of the observed gene set statistics. The calculation of the *p*-value can be done in three different ways:

**1. gene sampling**: A large number of random gene sets of the same size as the set under investigation is drawn from all the genes and the global statistic is recomputed for every random set. The *p*-value is calculated as the fraction of resampled gene set statistics that exceed (or fall below) the observed value.

**2. sample label permutation**: The phenotypes of the subjects are permuted a large number of times and the local and global statistics are recomputed. The *p*-value is the fraction of permutation gene set statistics that exceed (or fall below) the observed value.

**3. restandardization**: Both gene sampling and sample label permutation are conducted. Then each permutation statistic is standardized with the mean and standard deviation of all permutation statistics. To obtain the restandardized value, the standardized permutation statistic is multiplied with the standard deviation of the gene sampling statistics and the mean of the gene sampling statistics is added. The *p*-value is the fraction of restandardized gene set statistics that exceed (or fall below) the observed value.

Note that the choice of the null hypothesis and the pursued sampling strategy are interconnected: *Q*_1 _implies gene sampling, and *Q*_2 _sample label permutation. Furthermore, gene resampling implicitly assumes independent genes in the group, a prerequisite that is unlikely to hold, e.g., for genes within a pathway. The restandardization strategy put forward in [11] combines the two very different sampling schemes into a single procedure. Therefore, this approach mixes the two null hypotheses *Q*_1 _and *Q*_2 _which may lead to difficulties in the interpretation of the resulting *p*-values. Interestingly, note that the GSEA algorithm [7,8] implicitly also considers both permutation approaches. While the GSEA null distribution itself is found by permuting sample labels, the Kolmorogov-Smirnov-type test statistic equally carries information on the whole set of genes (at least if the set *S *is small compared to the number of all genes). Thus, GSEA implements something in effect related to restandardization, albeit not in the extent of the method by [11]. Finally, a computational consideration is whether the chosen null distribution may be approximated by a parametric distribution. This is fundamentally tied to the choices made earlier, and is possible only in very rare circumstances, e.g., in the method of Kim and Volsky [18].

#### Global and multivariate approaches

Enrichment approaches that do not follow the structure introduced above are the global and multivariate procedures. These directly define one model for the whole gene set. Nevertheless, the globaltest of [22] and the GlobalAncova of [23] also allow to obtain a score for every gene as well, since the gene set models can be written as a combination of the models for each gene. For the *T*^2^-based tests this is only possible when the covariance matrix is diagonal.

In a simulation study, [23] showed that the globaltest and their GlobalAncova perform equally well for independent genes but that the GlobalAncova outperforms the approach of [22] when there are strong gene-gene dependencies. Kong *et al. *[25] show that their combination of PCA and Hotelling's *T*^2^-test detects more gene sets than the globaltest. They explain this finding with the multivariate nature of their test. However, the assumption is only made on the basis of the evaluation of one data set and further analyzes are needed to show the superiority of this procedure.

## Results and discussion

Employing the developed taxonomy and modular framework we now compare the various gene set enrichment approaches by a principled simulation study and exemplary data analysis.

### Simulation study

#### General set-up

All necessary programming and calculations were implemented using the free software environment R [39]. We simulated data in a fashion to represent stylized yet typical constellations of gene set structures found in real gene expression data. We considered gene sets with different levels of differential expression (Δ*μ *= 0, 0.75, 1, -1) and with varying levels of intra-group correlation (*ρ *= 0, 0.6, -0.6). Furthermore, we also constructed mixed sets, i.e. gene sets that included both differentially expressed and non-differentially expressed genes, and also sets containing both up and down-regulated genes.

Each generated data set consisted of *p *= 600 genes with *n *= 20 samples (10 in each of two "treatment" groups). The data were generated from a 600-dimensional multivariate normal distribution, with variances set to 1 and means and correlations specified as follows:

• **background **(noninformative genes): 520 genes were simulated with *μ *= 0 and *ρ *= 0.

• **set 1 **(differential expression + correlation): 20 genes with Δ*μ *= 0.75 and pairwise correlation *ρ *= 0.6 between the genes in the set.

• **set 2 **(differential expression + no correlation): same as set 1 but with zero correlation among genes.

• **set 3 **(no differential expression, no correlation): 20 genes selected randomly from the 520 background genes.

• **set 4 **(differential expression + correlation, mixed with background): 10 genes with Δ*μ *= 0.75 and *ρ *= 0.6 from set 1, the other 10 randomly selected from the background set.

• **set 5 **(differential expression + no correlation, mixed with background): same as set 4, but with zero correlation among genes (i.e. 10 genes from set 2 and 10 genes from the background set).

• **set 6**: (differential expression, correlation, both up and down regulation): 10 genes with Δ*μ *= 1 and 10 genes with Δ*μ *= *-*1, the pairwise correlation between the genes with the same direction of differential expression is *ρ *= 0.6, between the up- and down-regulated genes it is *ρ *= -0.6.

• **set 7 **(differential expression, no correlation, both up and down regulation): same as set 6, but without any correlation among genes.

• **set 8 **(differential expression, correlation, both up and down regulation, mixed with background): 10 genes from set 6 (5 up-regulated and 5 down-regulated), mixed with 10 genes from the background set.

• **set 9 **(differential expression, no correlation, mixed with background, both up and down regulation): same as set 8, but without any correlation among genes (i.e. 10 genes from set 7 and 10 genes from background set).

Thus, the 600 simulated genes are constituted by the background set, set 1, set 2, set 6 and set 7. Any well working procedure for gene set enrichment analysis should be able to detect at least the pure sets 1, 2, 6 and 7, but ideally also the sets 4, 5, 8, 9 where only half of the genes are differentially expressed. Gene set 3 serves as a negative control.

Using this data we conducted gene set enrichment analysis by considering combinations of the following module methods:

• **gene-level statistics**: two-sample *t*-statistic, moderated *t*-statistic, Pearson correlation coefficient.

• **transformation of gene-level statistics**: none, squared values, ranks, binary transformations, local FDR.

• **gene set statistics**: mean, median, maxmean statistic, ES (weighted by absolute value of gene level statistics), conditional FDR (linear logistic regression of probability of gene set membership depending on the value of the gene-level statistic), Wilcoxon rank sum test.

• **significance assessment**: resampling, permutation, restandardization (1000 iterations each); parametric (only in combination with mean and Wilcoxon rank sum test).

The gene set statistic ES was not combined with a binary transformation since the latter does not allow a sensible ranking of the genes. Hence, in total 3 × 5 × 6 × 3 – 9 = 261 variants of gene set analysis were considered. For each combination of modules we simulated 100 data sets, and determined the false positive rate for set 3 and the true positive rates for the remaining sets.

#### Gene-level statistics and their transformations

First, we studied the effect of the choice of a specific gene level statistic and the corresponding transformation. Specifically, we varied the gene level statistic and the transformation and fixed the gene set statistic to the mean and computed *p*-values by resampling. The results are summarized in Table 2.

**Table 2:** The effect of choice of gene level statistic and of a corresponding transformation on the detection rate.

	**no transformation**			**squared**			**rank squared**		
	**t**	**mod.t**	**corr**	**t**	**mod.t**	**corr**	**t**	**mod.t**	**corr**
set 1	0.94	0.94	0.94	0.63	0.67	0.74	0.84	0.84	0.84
set 2	1.00	1.00	1.00	0.90	0.92	0.95	1.00	1.00	1.00
set 3	0.00	0.00	0.00	0.00	0.01	0.01	0.01	0.01	0.01
set 4	0.79	0.81	0.83	0.40	0.44	0.50	0.54	0.54	0.54
set 5	0.95	0.96	0.95	0.34	0.41	0.44	0.38	0.41	0.38
set 6	0.00	0.00	0.00	0.86	0.85	0.88	0.95	0.96	0.95
set 7	0.01	0.01	0.00	0.99	1.00	1.00	1.00	1.00	1.00
set 8	0.00	0.00	0.00	0.70	0.70	0.75	0.74	0.75	0.74
set 9	0.00	0.01	0.00	0.81	0.90	0.89	0.80	0.82	0.80

			
		**binary**			**local fdr**				
		**t**	**mod.t**	**corr**	**t**	**mod.t**	**corr**		
			
	set 1	0.62	0.61	0.55	0.49	0.48	0.46		
	set 2	0.81	0.87	0.73	0.77	0.78	0.71		
	set 3	0.01	0.00	0.01	0.00	0.00	0.00		
	set 4	0.38	0.44	0.34	0.32	0.36	0.32		
	set 5	0.27	0.33	0.20	0.23	0.26	0.20		
	set 6	0.83	0.85	0.76	0.71	0.66	0.69		
	set 7	1.00	1.00	0.99	0.99	0.99	0.95		
	set 8	0.63	0.66	0.58	0.61	0.60	0.55		
	set 9	0.71	0.73	0.65	0.74	0.75	0.66		

The values indicated are the proportion of significantly enriched gene sets (*p*-values ≤ 0.05) for various combinations of test statistics and their transformations, with fixed global statistic (= mean) and the use of resampling for computing the significance.

In general, the choice of the gene-level statistic does not seem to have a great impact on the results of the enrichment analysis. In our simulation setting we obtained roughly the same results, regardless whether the conventional or the moderated *t*-statistic or the correlation was used. We note that the small differences might be explained with the rather large number of samples per group (10) which is already sufficient to properly estimate the variance of each gene without using a regularization approach. For smaller sample sizes, the merits of regularization or borrowing information across genes is likely to be more pronounced. In contrast, we find that the choice of a transformation has quite a substantial effect on the detection rates. It is striking that all three investigated gene level statistics fail to find sets containing both up- and down-regulated genes. In this situation applying an appropriate transformation instead of using the original scores is indispensable. In this regard, a very useful transformation is the calculation of squared values. Although in Table 2 it can be seen that this results in slightly lower true positive rates for sets 1, 2, 4 and 5 compared to untransformed gene-level scores, it enables the detection of the sets 6, 7, 8, and 9, which are not recognized using the untransformed gene level statistics. Interestingly, if the squared transformation is combined with the rank transformation, this further improves the true positive rates and offers the best overall performance of all transformations under study (see Table 2). Furthermore, the (rank) quadratic transformations outperform the binary and the FDR transformations, which are two approaches that also detect sets containing both up and down-regulated genes.

#### Gene set statistics

Next, we considered in our simulations the impact of the choice of the gene set statistic. To facilitate comparison, we used as underlying gene level statistic the moderated *t*-statistic and employed a quadratic transformation. For significance assessment we applied the resampling. Table 3 shows the results.

**Table 3:** Impact of choice of gene set statistics on detecting gene set enrichment.

	**mean**	**maxmean**	**median**	**ES**	**cond. FDR**	**Wilcoxon**
set 1	0.67	0.67	0.82	0.56	0.68	0.84
set 2	0.92	0.93	0.98	0.65	0.94	1.00
set 3	0.01	0.01	0.00	0.00	0.01	0.01
set 4	0.44	0.45	0.57	0.35	0.45	0.54
set 5	0.41	0.41	0.32	0.22	0.42	0.41
set 6	0.85	0.86	0.94	0.80	0.85	0.96
set 7	1.00	1.00	1.00	0.99	1.00	1.00
set 8	0.70	0.71	0.78	0.65	0.84	0.74
set 9	0.90	0.86	0.81	0.69	0.99	0.80

The indicated values correspond to the proportion of *p*-values ≤ 0.05 for squared moderated *t *and resampling.

Overall, the gene set statistics all behave as expected for the nine sets. They assign high *p*-values to the uninteresting set 3 and relatively low *p*-values to the other sets. All approaches have some difficulty to reliably detect gene sets 4 and 5, where there is relatively weak differential expression and half of the genes belong to the uninformative background.

Performance-wise, the test statistics may be grouped as follows. The mean and the maxmean statistic produce rather similar and overall very good results. The median and the Wilcoxon rank sum test, which are coarsened and more robust location estimators for the change in transcript level, perform markedly better than the mean or maxmean for the correlated sets. However, if phenotype permutation instead of resampling is used, this effect vanishes and the performance of the median and the Wilcoxon test deteriorates (data not shown, cf. [40] for more information). Thus, the usage of the median and the Wilcoxon test is primarily advantageous if the competitive null hypothesis is tested, or if there are many outliers in the data. The conditional FDR procedure compares well to the mean and maxmean procedures in the simulations. However, we find that the results of this approach vary strongly with the choice of the gene-level statistic, transformation and permutation approach. Since it is not markedly better than the other approaches for any gene set and it is computationally expensive, it might not be the best overall choice for an enrichment analysis. Perhaps surprising is the comparatively weak performance of the popular ES score, which yields results that are worse than that of the sum or the mean.

#### Significance assessment

In a further simulation experiment, we compared four approaches for obtaining significance values (see Table 4). In this study we used moderated *t *as the gene level statistic, employed a quadratic transformation, and used the mean as the gene set statistic.

**Table 4:** Comparison of methods for assigning significance.

	**parametric**	**resampling**	**permutation**	**restandardization**
set 1	0.82	0.67	0.59	0.66
set 2	1.00	0.92	1.00	0.92
set 3	0.03	0.01	0.06	0.01
set 4	0.60	0.44	0.51	0.43
set 5	0.85	0.41	0.90	0.41
set 6	0.96	0.85	0.83	0.84
set 7	1.00	1.00	1.00	1.00
set 8	0.90	0.70	0.79	0.68
set 9	0.99	0.90	1.00	0.87

The values indicated are the proportion of *p*-values ≤ 0.05 using the gene set statistic mean of the individual squared moderated *t*-statistics.

First, the parametric approximation of the null distribution yields the best results. However, the assumption of independence of the individual genes is clearly violated for sets 1, 4, 6 and 8. Thus, the corresponding *p*-values are over-optimistic.

The resampling procedure that investigates the competitive null hypothesis *Q*_1 _also performs quite well. Sets 3 and 7 are correctly categorized for almost all the 100 repetitions of the simulation. For sets 2 and 9 this is achieved in more than 90% of the cases. The correlated sets 1, 6 and 8 achieve lower true positive rates. Resampling performs worst for the difficult sets 4 and 5, where only half of the genes are differentially expressed.

Testing the self-contained null hypothesis *Q*_2 _using sample label permutation appears to be slightly easier than testing *Q*_1 _(see third column of Table 4), especially for the uncorrelated gene sets. Note that permutation preserves the correlation structure among genes. Since the effective gene set size is smaller in a correlated set compared to a set where the genes are independent, it is more difficult to detect enrichment in these situations. For this reason it is surprising that in most of the cases the permutation works still better for the correlated sets than resampling.

The restandardization procedure performs very similar to resampling. This was the case in almost all of the combinations of enrichment methods that were investigated in this work, so the benefits of restandardization are likely to be small in general. However, we note that [11] showed in their paper that there might be some special situations where restandardization is advantageous.

#### Comparison with global and multivariate approaches

In order to compare the modular univariate approaches with the global and multivariate procedures, we additionally analyzed the simulated data using the globaltest and Hotelling's *T*^2^-test with a shrinkage covariance matrix [41].

The results for the globaltest under different significance assessment approaches are summarized in Table 5. Clearly, the performance of this procedure is not better than that of the less sophisticated univariate methods, especially the sum or mean of the squared gene-level statistics. Indeed, the results are very close to these two approaches. Hence, using the globaltest does not improve the results, but it is computationally a little bit faster, especially when the parametric approximation of the null distribution is used. Since it leads to comparable results, it might be a useful alternative when a large number of sets is to be investigated.

**Table 5:** Performance of the globaltest.

	**parametric**	**resampling**	**permutation**	**restandardization**
set 1	0.59	0.66	0.61	0.63
set 2	1.00	0.94	1.00	0.93
set 3	0.02	0.00	0.05	0.00
set 4	0.46	0.44	0.49	0.44
set 5	0.85	0.42	0.91	0.43
set 6	0.82	0.85	0.80	0.84
set 7	1.00	1.00	1.00	1.00
set 8	0.74	0.69	0.76	0.68
set 9	0.99	0.86	1.00	0.89

The indicated values are the proportion of *p*-values ≤ 0.05.

The corresponding results for the Hotelling *T*^2^-test can be found in Table 6. Using this approach the uncorrelated sets are found with the same reliability as with univariate approaches. However, in stark contrast, the sets with correlation (sets 1, 4, 6, and 8) are hardly detected. The reason is clear from a statistical perspective: the Hotelling test statistic penalizes correlation (i.e. the test statistic is largest for zero correlation). Specifically, it is much more likely that genes in a set are all simultaneously differentially expressed if there is correlation. While the Hotelling statistic corrects for this bias accordingly, this implicit penalization is clearly not desired from a biological point of view. A clear advantage of the Hotelling *T*^2^-test is its improved performance with sample label permutation as opposed to gene sampling. Since the approach does by definition test the self-contained null hypothesis, this is a positive result. Nevertheless, the overall very poor performance of this multivariate approach raises the question whether taking into account the dependence structure between the genes is at all useful in the context of enrichment analysis.

**Table 6:** Performance of the Hotelling approach using a shrinkage correlation matrix.

	**resampling**	**permutation**	**restandardization**
set 1	0.09	0.18	0.08
set 2	0.92	1.00	0.92
set 3	0.01	0.05	0.01
set 4	0.06	0.16	0.05
set 5	0.41	0.91	0.40
set 6	0.25	0.26	0.22
set 7	1.00	1.00	1.00
set 8	0.25	0.51	0.25
set 9	0.89	1.00	0.87

The indicated values are the proportion of *p*-values ≤ 0.05.

### Analysis of p53 cancer data

In order to verify our conclusions from the simulations we reanalyzed the p53 cancer data from Subramanian *et al. *[8]. The protein p53 is a tumor suppressor preventing the development of cancer cells. It regulates genes involved in the cell cycle and the induction of apoptosis after DNA damage. Gene set enrichment analysis allows to study which pathways are involved in these cellular mechanisms.

The p53 data is used as a benchmark data set in a number of papers [e.g., [19,11]] and contains expression profiles of 50 cancer cell lines that can be found in the data base of the International Agency for Research in Cancer (IARC). The gene expressions were measured with the Affymetrix HGU95Av2 chip which contains 12, 625 probe sets. For each cell line, the mutational status of the p53 protein was reported. 17 cell lines were found to have normal p53 status while the remaining 33 samples showed a mutation of p53. The analyzes in [8,19] and [11] recovered several gene sets that are differentially expressed between the mutation and wildtype cell lines. Five functional groups were mentioned in all three publications:

• the p53 pathway,

• the down-stream targets of p53,

• genes induced by radiation,

• genes induced by hypoxia, and

• the heat-shock protein signaling pathway.

Dinu and co-workers presented 31 additional gene sets found with the SAM-GS method [19].

In our preprocessing of the data we filtered out genes that showed maximum signal intensities of less than 5 on log_2 _scale and a variation of less than 2-fold over all samples to reduce the noise in the data. The resulting data set contained 8,768 genes. From the biologically chosen gene sets of [8] we focused on sets with sizes between 15 and 500 genes. This left 290 gene sets of varying size, each containing between 15 and 321 genes.

In the following we report only our main findings. A much extended analysis of the p53 data set including detailed tables of significant gene sets and pathways can be found in [40].

#### Gene-level scores and transformations

As in the simulation study, in order to compare the various gene level statistics and their transformations we employed as gene set statistic the mean and use resampling for computing *p*-values.

Concerning the choice of gene statistics, there was little difference in the overall ranking of the gene sets. The 25 top ranked gene sets are nearly the same for all three statistics in combination with a specific transformation. The significant findings are in broad accordance with the results published previously [8,19,11]. Specifically, we also recover the additional sets reported by Dinu *et al. *[19] if a squared gene transformation is used (recall that their SAM-GS approach is based on a squared regularized *t-*statistic). For this data set we noted a distinct difference when positive and negative scores were analyzed separately, instead of combined. In particular, taking squared values of the individual gene scores appears to give more weight to gene sets that are not high scored in a separate analysis for up- and down-regulated genes. This is probably due to the fact that summing up the squared values leads to the detection of gene sets with many subtle expression changes in both directions instead of sets where some constituents are either up- or down-regulated.

When ranks or the binary transformation are used, fewer of the Dinu and Subramanian gene sets are detected. Furthermore, the number of significant gene sets is decreased compared to using the original or squared scores. Thus, for this data the results based on the rank and binary transformations appear to suffer substantially due to the loss of information. In contrast, the transformation to local FDR values (empirical posterior probabilities) performs much better than in the simulation study.

#### Gene set statistics and significance assessment

Next, we investigated the impact of the gene set statistic and the choice of the significance procedure. In this analysis we used the squared moderated *t*-statistic as the gene level statistic.

**Figure 2:**
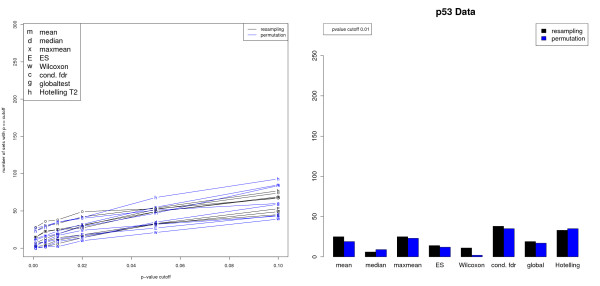
**Analysis of p53 data set**. Left: Number of significant gene sets in dependence of  *p*-value cutoff and choice of gene set statistic. On the gene level, the squared moderated *t*-statistic was employed. Right: Bar plot for *p*-value cutoff 0.01.

**Figure 3:**
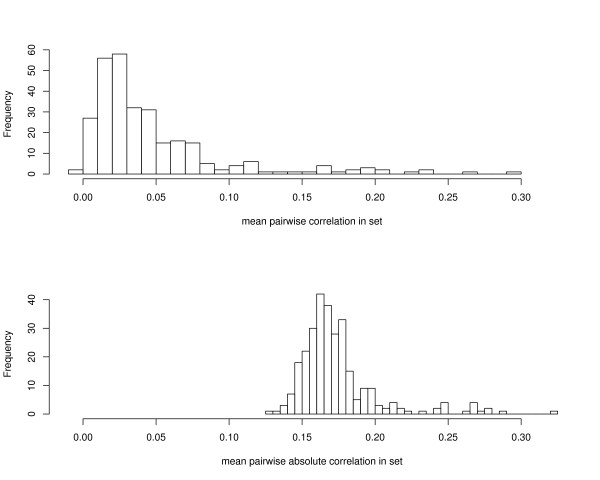
**Distribution of correlation across the 290 gene sets investigated for the p53 data**. Top: histogram of averaged pairwise correlations. Bottom: histogram of averaged absolute values.

The results are summarized in Fig. 2. For this data, both phenotype permutation as well as resampling lead to the same number of significant gene sets regardless of the *p*-value cutoff, the only exception being the Wilcoxon statistic. The Hotelling statistic and the conditional FDR approach consistently found the largest number of significant gene sets for any cutoff, followed by the global test and the mean and maxmean. The ES, median, and Wilcoxon statistics typically resulted in the least number of gene sets. Note, however, that we do not know the ground truth for the p53 data. Therefore, one needs to keep in mind that the larger power of the mean or the global test compared, e.g., to the ES score might be due to a higher specificity of the latter. This is in concordance with our simulation study (cf. Table 3) where ES and the median were the only two statistics that never declared the negative control (set 3) significant. The outcome of the Hotelling *T*^2^-test is in very good accordance with the findings of [19]. Thus, in contrast to the simulated data the Hotelling approach gives meaningful results for real data. We investigated this discrepancy more closely by inspecting the correlations among the genes in a gene set. As can be seen from Fig. 3 the correlations within a gene set are comparatively weak. This explains the good result of the Hotelling approach, which for vanishing correlation essentially reduces to the SAM-GS [19].

### Analysis of Hedenfalk breast cancer data

To complement the study of gene expression data we analyzed the breast cancer gene expression data from Hedenfalk *et al. *[42]. This well-studied data set includes 7 cancer patients carrying the BRCA1 genetic mutation and 8 other patients carrying the BRCA2 mutation. Microarray measurements were taken on 3,226 genes.

A peculiar feature of this data set is that on the gene level there is virtually no signal to distinguish the two groups (BRCA1 versus BRCA2) – see for example [43].

In our preprocessing for gene set analysis we considered a subset of 2,200 genes (all that could be uniquely mapped from cloneIDs to a unigene symbol) and a catalog of 89 gene sets from [8] relevant for these genes.

#### Gene set statistics and significance assessment

**Figure 4:**
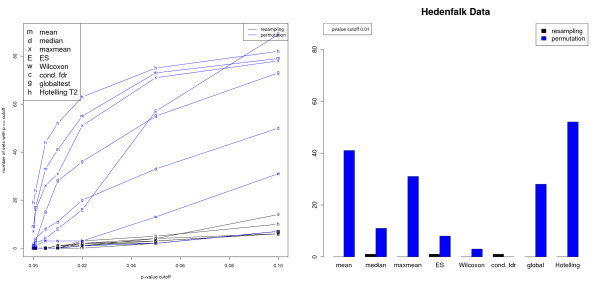
**Analysis of Hedenfalk data set**. Left: Number of significant gene sets in dependence of *p*-value cutoff and choice of gene set statistic. On the gene level, the squared moderated *t*-statistic was employed. Right: Bar plot for *p*-value cutoff 0.01.

The p53 and the Hedenfalk cancer data behave strikingly different, as can be seen in Fig. 4. For the Hedenfalk data employing resampling resulted in very few gene sets detected as significant, regardless of gene set statistic. In contrast, when phenotype permutation was applied a substantial number of significant gene sets were found. As for the p53 data set, the gene set statistics can be divided into two groups. The Hotelling, mean, maxmean and global statistics resulted in larger numbers of significant set, whereas median, ES, Wilcoxon and conditional FDR detected fewer gene sets. Thus, for this data the conditional FDR approach is as conservative as in the simulations.

The difference between results for permutation and resampling may be explained as follows. If most of the active genes show only small expression changes, then the interesting sets do not differ markedly from random sets. Consequently, they cannot be found by the resampling approach. But still, in the Hedenfalk data the subtle changes of the interesting genes between the phenotypes are large enough when they are accumulated over the active gene set to be detected with sample label permutation.

#### Enriched gene sets

Finally, it is also instructive to inspect the list of detected gene sets. For gene set statistic mean with gene-level statistic moderated *t*, transformation squared values and sample label permutation all significant (*p*-value cutoff 0.01) gene sets are listed in Table 7. Note the enrichment of many cancer-related sets as a result of the gene set analysis.

It is noteworthy here that using the ES as gene set statistic does not yield the set Breast_Cancer_Estrogen_Signalling among the 20 top ranked gene sets, whereas in our Table 7 it is ranked number 1. We remark that this is a biologically important finding, in concordance with Hedenfalk *et al. *[42] who state that tumors with BRCA1 mutations are generally negative for estrogen receptors, whereas BRCA2 mutations are positive, implying that BRCA1 and BRCA2 genes induce the formation of breast tumors through separate pathways.

**Table 7:** Top scoring gene sets resulting from the analysis of the Hedenfalk data.

	Gene set	p-value
1	breast_cancer_estrogen_signalling	0.000
2	cell_surface_receptor_linked_signal_transduction	0.000
3	insulin_signalling	0.000
4	p53_signalling	0.000
5	pparaPathway	0.000
6	VOXPHOS	0.000
7	RAP_UP	0.000
8	PROLIF_GENES	0.000
9	UPREG_BY_HOXA9	0.000
10	cell_adhesion	0.001
11	CR_CAM	0.001
12	CR_DEATH	0.001
13	il2rbPathway	0.001
14	ST_Tumor_Necrosis_Factor_Pathway	0.001
15	tcrPathway	0.001
16	HTERT_UP	0.001
17	CBF_LEUKEMIA_DOWNING_AML	0.001
18	CR_SIGNALLING	0.002
19	ghPathway	0.002
20	ST_B_Cell_Antigen_Receptor	0.002
21	tpoPathway	0.002
22	GO_0005739	0.002
23	LEU_UP	0.002
24	Cell_Cycle	0.003
25	GLUT_UP	0.003
26	FRASOR_ER_DOWN	0.003
27	ANDROGEN_UP_GENES	0.003
28	fmlppathway	0.004
29	hivnefPathway	0.004
30	biopeptidesPathway	0.005
31	cell_adhesion_molecule_activity	0.005
32	SIG_InsulinReceptorPathwayInCardiacMyocytes	0.005
33	ST_Integrin_Signaling_Pathway	0.005
34	drug_resistance_and_metabolism	0.006
35	ST_Differentiation_Pathway_in_PC12_Cells	0.007
36	gleevecPathway	0.008
37	DNA_DAMAGE_SIGNALLING	0.009
38	ST_ERK1_ERK2_MAPK_Pathway	0.009
39	mRNA_splicing	0.010
40	nfatPathway	0.010
41	HUMAN_CD34_ENRICHED_TF_JP	0.010

As gene set statistic we used the mean combined with the squared moderated *t-*statistic on the gene level and sample label permutation.

Overall, it appears that the Hedenfalk data is an exemplary case where merits of gene set enrichment over analyzing individual genes can be most prominently seen.

## Conclusion

In this work we have investigated statistical procedures for detecting enrichment of gene sets. By conducting an extensive survey of corresponding approaches we found that all procedures in current use may be fit into a simple modular framework as depicted in Fig. 1.

The identification of this modular structure greatly facilitates the systematic comparison of the diverse methods. Based on simulations and data analysis we arrive at a number of guidelines for conducting a gene set enrichment analysis. We believe that these will contribute to the establishment of best practices for gene set enrichment analysis.

Perhaps the most surprising result from our study is that the simple univariate procedures appear to work best under a large variety of conditions. Specifically, using the mean of the squared (regularized) *t*-statistics and assessing the significance with one of the two permutation approaches reliably detects gene sets of diverse types of expression and correlation structures. This procedure was proposed, e.g., in [15,14,19,17]. Intriguingly, this simple approach typically outperforms (by a fairly large margin) the in biological circles very popular GSEA method [8]. Thus, we strongly disagree with the recommendation put forward in [34] which was primarily given on grounds of software availability.

With regard to the choice of the best approach for each module in Fig. 1, our conclusions can be summarized as follows:

• **gene-level score: **The choice of the gene-level score is not crucial. All different kinds of scores lead to very similar results. However, it is recommended to use a statistic that is standardized by the variation of the gene expression, as this makes the measure of differential expression comparable across genes. Scores with this characteristic are *t*-statistics, correlation coefficients or standardized regression coefficients. If the sample size is small, using one of the regularized *t*-statistics is the best choice since they are designed to stabilize small variances.

• **transformation: **The choice of transformation has a substantial impact on the overall results. In our study the (rank) squared transformation was most accurate. The application of absolute values instead of squared values gives comparable results. Note that using the squared values essentially corresponds to using a modified version of Hotelling's *T*^2^-test with a diagonal covariance matrix. Binary transformations often lead to satisfying results but also imply a loss of information. If the focus is put on gene sets that are concertedly regulated in one direction the untransformed scores should be used.

• **gene set statistics: **Overall, the simple approaches such as the mean or the median work very well. Using the median instead of the mean was proposed by [16] as an approach that is less sensitive with regard to outliers. However, using the median may also lead to a smaller number of significant findings. Similar arguments apply to the rank-based Wilcoxon test. The GSEA, or more precisely the enrichment score, is not as reliable as the other gene set test statistics. Furthermore, the construction of the GSEA is rather complicated since it mixes the competitive and the self-contained null hypotheses.

• **significance assessment: **The choice of the permutation approach for significance assessment is mainly determined by the null hypothesis that is tested. Hence, it has to be decided carefully which procedure is used. If a self-contained test is conducted, the phenotypes have to be permuted. In this way, the null distribution of the gene set statistic under the assumption of no difference between the treatment groups is obtained. If the gene set is to be compared to other sets, gene sampling is the correct approach. Then the null model is based on the assumption that the gene set membership is random. Depending on the data structure, both approaches can yield quite different results.

• **multivariate procedures: **Using a multivariate procedure such as the globaltest or the Hotelling approach not necessarily yields better results than the best univariate approaches. However, for the globaltest a parametric approximation of the null distribution is available, which might be beneficial if a large number of gene sets are analyzed. The Hotelling-type approach is not appropriate for the detection of gene sets containing genes that are highly correlated. From a statistical perspective this is straightforward to understand, as it is more unexpected to find a group of independent genes that are co-regulated than a group of correlated genes which behave similarly.

In conclusion, we find that despite the large variety of available gene set enrichment procedures there is a common core among all procedures. In order to reliably detect enriched gene sets it is in general sufficient to use a univariate approach, in combination with either resampling or sample label permutation, depending on the desired underlying null model.

In the present study, we have focused on methods for detecting enrichment of a single prespecified gene set. A topic not investigated here is the issue of multiple testing of gene sets. In this context, standard corrections for multiplicity need to be refined in order to account for dependencies among gene sets, which may be due to the underlying gene ontology graph structure or because genes may belong to several gene sets at once [44,28].

## Authors' contributions

Both authors contributed equally to the development of the modular framework, the design of the study, and to writing the paper. MA conducted all simulations and data analysis.

## Appendix

In this section we provide an overview of various currently employed statistical methodologies for detecting enrichment of gene sets. We distinguish between univariate and multivariate approaches. The univariate procedures first calculate a score for differential expression for each single gene. Subsequently, a gene set summary statistic is computed and its significance is assessed by different parametric and permutation approaches. In contrast to the univariate approaches, the global and multivariate procedures directly infer the enrichment of the gene set without referring back to the gene-level. The multivariate approaches are distinguished from the global procedures by explicitly taking the correlation matrix of the genes into account when calculating the test statistic

### Univariate procedures

#### Overrepresentation analysis

One of the simplest methods to investigate enrichment of predefined gene sets is based on testing a 2 × 2 contingency table. Correspondingly, this approach has been employed by many authors, e.g. [1,4]. In this setting, each gene is classified according to both membership in a gene set *S *and whether it is differentially expressed (cf. Table 8). Subsequently, one investigates overrepresentation of genes belonging to *S *in the group of the differentially expressed genes. This is done by testing for the independence of the two criteria "differential expression" and "gene set membership". For instance, one can use the test statistic

**Table 8:** Contingency table for testing gene set enrichment.

	**differentially expressed**	**non-differentially expressed**	**total**
**in gene set**	*n*_11_	*n*_12_	*n*_1_.
**not in gene set**	*n*_21_	*n*_22_	*n*_2_.

**total**	*n*._1_	*n*._2_	*N*

$$\chi^{2}=\frac{N{\left(\left|n_{11}n_{22}-n_{12}n_{21}\right|-\frac{N}{2}\right)}^{2}}{n_{1}.n_{2}.n._{1}n._{2}},$$

which for sufficiently large *N *follows a *χ*^2^-distribution with one degree of freedom. Alternatively, one may compute Fisher's exact test [45] for the hypergeometric distribution [2,3]. It is also possible to determine the distribution of the test statistic nonparametrically using sample label permutation [5], which has the benefit of respecting the correlation between the genes. Vêncio and Shmulevich [6] introduce an approach to account for categorization uncertainty, employing the Goodman-Kruskal [46] measure of statistical association between the two margins of the contingency table.

#### GSEA algorithm and related methods

One of the most popular methods for detecting enrichment is the "Gene Set Enrichment Analysis" (GSEA) introduced in [7] and improved by the same group only a short time later [8].

This method starts with a list *L *of *N *genes ranked by their correlation with the phenotype of interest, e.g., using the *t*-score or the signal-to-noise ratio. In order to test the null hypothesis that the *m *genes in a set *S *are randomly spread in the list *L *a so-called enrichment score (*ES*) is computed. This equals the maximum deviation from zero of a running sum (running down the list of genes *L*) that increases every time a gene in the list *L *is in *S *and decreases every time a gene is not in *S *(*j *= 1, ..., *N*). Additionally, every gene set member is weighted by its absolute value of correlation with the phenotype. As a result, genes with very low correlations do not contribute to the enrichment score of the set. The running sums for the genes in the set *S *and the complementary set $\bar{S}$ (including all the genes from the list which are not in *S*) are calculated separately as follows:

$$\begin{array}{ll} ES_{S}(l)=\sum_{\begin{array}{c} g_{j}\in S\\ j\leq l \end{array}}\frac{|r_{j}{|}^{p}}{N_{S}}, & \text{where }N_{S}=\sum_{g_{j}\in S}|r_{j}{|}^{p},\\ ES_{\bar{S}}(l)=\sum_{\begin{array}{c} g_{j}\notin S\\ j\leq l \end{array}}\frac{1}{N-m}. & \end{array}$$

*r*_*j *_is a measure for the correlation of the individual gene *g*_*j *_with the phenotype, for example a *t*-statistic. The *ES *is defined as the maximum deviation of *ES*_*S *_(*l*) - $ES_{\bar{S}}(l)$ from zero (*l *= 1, ..., *N*). This test statistic is similar to a Kolmogorov-Smirnov statistic in that it compares the deviation between two cumulative distribution functions. But contrary to the Kolmogorov-Smirnov statistic, the *ES *is signed and uses weights.

The choice of the exponent *p *in the above formula determines the influence of the single genes on the score. For *p *= 0 an unweighted statistic is obtained. When *p *= 1 the genes in *S *are weighted by their absolute value of correlation with the phenotype according to the chosen correlation measure. Other values of p can also be used (see [8], supplementary material). The authors suggest *p <*1 if one wishes to penalize a lack of coherence in a set or *p > *1 if correlation of a small subset of *S *with the phenotype is sufficient to call a set enriched. Additionally, the summands are normalized by the sum of all test statistics in *S*. The summands for the genes in $\bar{S}$ are constant.

Other authors presented similar statistics, see for example [12] who use *X*_*j *_= *N *- *m *and *X*_*j *_= -*m *as the contribution of the genes in *S *and $\bar{S}$ to the running sum respectively. All the choices result in a score that assumes the value 0 when the running sum reaches the end of the list *L*.

Significance of the test statistics is assessed by permuting the class labels and recalculating the *ES *for every gene set in every permutation. This necessitates correction for multiplicity when a larger number of sets is tested.

An approach related to GSEA but more general is "Significance Analysis of Function and Expression" (SAFE) as described by [9]. This is a two-step procedure, where in the first step a local test statistic is computed for each gene (e.g. a *t*-score) and in the second the statistics of the genes in the gene set *S *are combined to give a global statistic. This is done in order to compare the distribution of the local statistics in the gene set with that of the local statistics of the rest of the genes. However, [9] leave it to the user to decide which global statistic is to be applied. They propose rank-based statistics as the Wilcoxon rank sum test or the Kolmogorov-Smirnov test since they do not require any assumptions about the joint distribution of the local statistics. Significance in the SAFE algorithm is evaluated by permuting the sample labels and recalculating the local and global statistics. For taking account of multiplicity when testing for several gene sets at a time [9] suggest to control the false discovery rate and related criteria. 

Yet another modification of the GSEA method is introduced in [10]. These authors employ regression coefficients of the interesting outcome (in their case the age of the patient) to rank the genes. Instead of taking a Kolmogorov-Smirnov statistic as the gene set statistic, [10] use a van der Waerden statistic

$$Y=\sum_{g_{j}\in S}\Phi^{-1}\left(\frac{r_{j}}{N+1}\right),$$

where Φ^-1 ^is the quantile function of the standard normal distribution *N *(0, 1) and *r*_*j *_is the rank of gene *g*_*j *_in the list of all genes. Under the null hypothesis the test statistic *Y *is approximately normally distributed with mean zero. [10] employ a bootstrap sampling approach to estimate the variance of the null distribution.

#### Averaging methods

Another natural approach of combining information of a group of genes pursued by many authors is to simply average over the test statistics of the individual genes. Specifically, let *t*_*j*_, *j *= 1, ..., *N*, be the association measure of gene *g*_*j *_with the phenotype. Furthermore define *G*_*kj *_as the binary indicator of whether gene *g*_*j *_belongs to gene set *S*_*k *_(*G*_*kj *_= 1) or not (*G*_*kj *_= 0). Then the test statistic is given by

$$W_{k}=\frac{1}{m_{k}}\sum_{j=1}^{N}G_{kj}t_{j},$$

where $m_{k}=\sum_{j=1}^{N}G_{kj}$ is the number of genes in set *S*_*k*_.

A crucial point noted by [14] is that one needs to distinguish between two types of null hypotheses, which in turn necessitates different ways of assigning significance. The two hypotheses are:

1.*Q*_1_: "The genes in a gene set show the same pattern of association with the phenotype compared with the rest of the genes."

2. *Q*_2_: "The gene set does not contain any genes whose expression levels are associated with the phenotype of interest."

These correspond to the "competitive" and "self-contained" hypotheses discussed in [5]. Note that the first hypothesis compares the genes in the set with the remaining genes, thus implying that in a permutation test the genes are the sampling units. *Q*_2 _tests the association between the genes in the set and the phenotype regardless of the genes that are not in the set. Therefore, here the phenotype labels are the sampling units. When testing *Q*_2 _[14] employ a permutation approach whereas for testing *Q*_1 _they apply a normal approximation to compute *p*-values.

An averaging approach based on the moderated *t-*statistic is described in the documentation of [15]. Further discussion regarding the averaging of gene-level statistics can be found in [20,33] and [18]. The "category" software of [17] also implements a general form of the averaging approach.

#### The maxmean statistic and restandardization

[11] propose several improvements of the averaging approach and GSEA. First, they introduce a new test statistic called the "maxmean" statistic which leads to more effcient tests. It is defined as

$$T_{\text{maxmean}}=\max\left({\bar{s}}_{S}^{\left(+\right)},{\bar{s}}_{S}^{\left(-\right)}\right),$$

where ${\bar{s}}_{S}^{\left(+\right)}=\frac{1}{m}\sum_{g_{j}\in S}s_{j}^{\left(+\right)}$ and ${\bar{s}}_{S}^{\left(-\right)}=\frac{1}{m}\sum_{g_{j}\in S}s_{j}^{\left(-\right)}$ are the averages of the positive and negative parts of the scores in the set *S *containing *m *genes. The separation of positive and negative scores facilitates the detection of gene sets containing both up- and down-regulated genes. Furthermore, the form of the test statistic prevents that sets with only very few unusually large *z*-values are called significant, yet it allows to detect sets with both moderately large positive and negative *z*-values.

Second, [11] show the necessity not only to incorporate sample label permutation as in the GSEA method but also gene sampling into the analysis. This idea is implemented in a procedure called "restandardization" which combines gene sampling and phenotype permutation.

Let *μ*^† ^and *σ*^† ^be the mean and standard deviation of the distribution obtained by gene label permutation and μ* and σ* the corresponding parameters of the sample label permutation distribution. For a general test statistic *T*, for example the ES used in GSEA, the restandardization can be written as

$$T^{**}=\mu^{†}+\frac{\sigma^{†}}{\sigma^{*}}\frac{T^{*}-\mu^{*}}{\sqrt{m}}.$$

It corrects the phenotype permutation statistics for the estimated distribution of the individual genes in the data set. The corresponding *p*-value can then be calculated in the usual way as the fraction of a large number of restandardized test statistics exceeding the observed test statistic.

When the gene set score is simply the average of the individual gene scores in the set, the restandardization formula can be simplified to

$$T_{\text{ave}}^{**}=\mu_{s}+\sigma_{s}\frac{T_{\text{ave}}^{*}-\mu^{*}}{\sigma^{*}}.$$

Here *T*_*ave *_denotes the gene set statistic obtained by an averaging approach, *μ*_*s *_and *σ*_*s *_are the mean and standard deviation of the scores *s*_*j *_of all *N *genes and μ* and σ* are the mean and standard deviation of the gene set test statistics $T_{\text{ave}}^{*}$ obtained by sample label permutation. In this special case, it is not necessary to conduct gene sampling before doing the restandardization since the mean of the gene sampling distribution is the same as the overall mean of the single genes and the standard deviations only differ by the factor $\frac{1}{\sqrt{m}}$.

#### Extensions to GSEA and averaging methods

Some further variations to the GSEA and methods using the average of gene-level statistics are reported in Jiang and Gentleman [16]. They propose using the median or a sign test for robustly averaging over the scores in a gene set. [16] also suggest an alternative test statistic for the individual genes when there are other covariates such as gender or age which might influence the expression levels. In essence, this test statistic is based on fitting a linear model where the gene expression is the response and the phenotype and other covariates are the independent variables.

A similar approach was proposed by [13] who additionally incorporate a measure for co-expression in the gene sets, the mean of the pairwise correlation coefficients, and a measure for the distinctiveness of the sets based on a nearest neighbors classifier.

In their paper, [16] also propose a Bayes approach for the investigation of gene set enrichment. The procedure is somewhat similar to the two groups empirical Bayes model described in [21]. Another issue addressed by [16] is the potential overlap of gene sets. They also investigate dimension reduction (i.e. principle components analysis) to find gene sets with co-regulated genes.

#### Parametric method

Another very simple approach to detecting gene set enrichment is PAGE (Parametric Analysis of Gene Set Enrichment) suggested by [18]. Essentially, this approach proceeds by averaging over fold changes or other gene-level statistics and comparing this test statistic with the standard normal distribution. Since the approach is fully parametric and because no permutation is required this procedure is very time saving and computationally efficient. Using the normal approximation is justified by [18] via asymptotic arguments. In a similar fashion, the "category" averaging approach [17] also employs (among other options) a normal approximation for significance assessment.

#### Random-set method

Newton *et al. *[20] aggregate some ideas from the averaging methods, the parametric models, and the 2 × 2 table methods. Instead of only counting the number of differentially expressed genes present in the gene set, as done when applying 2 × 2 table methods, they allow for more general test statistics similar to the ES introduced by [7]. However, they do not apply sample label permutation to assess significance but consider the genes to be the sampling units.

Starting with the measures of differential expression *s*_*j*_, *j *= 1, ..., *N *for each gene, an unstandardized score for a gene set *S *consisting of *m *genes is calculated as the average of the gene scores of these genes

$$\bar{X}=\frac{1}{m}\sum_{g_{j}\in S}s_{j}.$$

As the method aims to compare the enrichment in a gene set *S *with the enrichment of all other $\left(\begin{array}{c} N\\ m \end{array}\right)$ distinct randomly drawn gene sets of size *m*, a so called random-set model is introduced. That means that the gene set *S *is now considered as a random collection of *m *genes whose scores *s*_*j *_are fixed. For most choices of the gene-level statistic, the exact distribution of $\bar{X}$ becomes intractable. [20] propose to approximate it with the normal distribution with mean and variance as follows:

$$\begin{array}{c} \mu =\text{E}(\bar{X})=\frac{1}{N}\sum_{j=1}^{N}s_{j}\\ \sigma^{2}=\text{Var}(\bar{X})=\frac{1}{m}\left(\frac{N-m}{N-1}\right)\left[\left(\frac{1}{N}\sum_{j=1}^{N}s_{j}^{2}\right)-{\left(\frac{1}{N}\sum_{j=1}^{N}s_{j}\right)}^{2}\right]. \end{array}$$

The score can then be standardized with these two quantities, resulting in the test statistic

$$Z=\frac{\bar{X}-\mu }{\sigma }.$$

Under the null hypothesis of no enrichment, *Z *is approximately normally distributed with mean zero and unit variance.

#### A false discovery rate-based method

Another approach to enrichment analysis is presented by [21]. This procedure is based on estimating the false discovery rate (FDR) of a gene conditional on belonging to a given gene set. In this analysis two subgroups of genes are considered – the genes in the gene set and the rest of the genes – and a mixture model for computing false discovery rates is fitted separately to these two subgroups. In addition, the false discovery rate is also computed without consideration of subgroups.

Efron [21] showed that the conditional local FDR and the unconditional local FDR are linked by the simple relation

$${\text{fdr}}_{S}(z)=\text{fdr}(z)\cdot \frac{\pi_{S0}(z)}{\pi_{S}(z)},$$

where *π*_*S*0_(*z*) is the probability for a null case of being in *S *given that its *z*-value is equal to *z*. *π*_*S *_(*z*) is defined analogously.

In this framework the null hypothesis of no enrichment corresponds to the statement that fdr_*S *_(*z*) is equal to the overall fdr(*z*), implying that the genes in *S *do not behave differently compared to all the *N *genes together. If the set is enriched, then fdr_*S *_(*z*) will be smaller than fdr(*z*) for very large (or small) *z*-values indicating differential expression in the set. Note that this assumes that the densities for the non-expressed genes are the same for the genes in *S *and those in $\bar{S}$ (i.e. *f*_*S*0_(*z*) = $f_{\bar{S}0}$(*z*)). [21] describes a corresponding test based on logistic regression.

### Global and multivariate procedures

#### Globaltest

Goeman *et al. *[22] approach the problem of enrichment analysis from the perspective of class prediction. They point out that if the gene set is associated with the phenotype, then its constituents will have different expression patterns in the two types of the response and thus will serve as reliable predictors for the clinical outcome. Testing for enrichment can therefore be done in the framework of generalized linear models. Let ***Y ***be the vector of the phenotypes of the *n *samples. For simplicity it is assumed that ***Y ***only takes the two values 0 and 1 for the two phenotypes although in this setting ***Y ***could also be continuous, for example the survival time of a patient. Furthermore, let ***X ***= (*x*_*ij*_) be the *n *× *m *matrix of expression patterns of the genes, where *m *is the number of genes in the gene set under investigation and *n *is the number of samples. In a generalized linear model, the influence of the gene expression on the phenotype can be modeled as

$$\text{E}(Y_{i}|\beta )=h^{-1}(\alpha +\sum_{j=1}^{m}x_{ij}\beta_{j}),$$

where *β*_*j *_is the regression coefficient of gene *g*_*j*_, *α *is the intercept and *h *is the link function. This can for example be the identity or the logit function, resulting in a simple linear model or a logistic regression model respectively. If the set is not enriched, then the genes do not have a predictive potential. Hence, a null hypothesis for the enrichment problem is

*H*_0 _: *β*_1 _= *β*_2 _= ... = *β*_*m *_= 0.

However, the size of the gene set may be quite large, so that *m *is greater than *n*. In this situation, the hypothesis above cannot be tested in the usual way. The solution proposed by [22] is to assume that the regression coefficients all follow the same distribution with mean zero and a common but unknown variance *τ*^2^. Then the test problem can equivalently be expressed as

*H*_0 _: *τ*^2 ^= 0.

Goeman *et al. *[22] develop a corresponding score test called "globaltest". It is particularly powerful for alternative hypotheses where all the *β*_*j *_are not equal to zero but do not deviate too much from the null hypothesis either. Thus, the globaltest seems very useful with regard to the underlying biological question about whether the set of genes as a whole influences the phenotype, i.e. whether or not a large proportion of the genes in the set have a moderate influence on the response.

We note that the globaltest is implemented assuming a diagonal covariance matrix. This means that the genes are considered to be uncorrelated and the test statistic can therefore be expressed as the average of the gene-wise test statistics for the individual genes. Thus, the method is comparable to the averaging procedures that first calculate a score for each gene and then summarize these scores for each set.

#### GlobalAncova

The linear model can also be used for enrichment analysis when the roles of gene expression and phenotype are exchanged. This was shown by Mansmann and Meister [23]. The authors call their method GlobalAncova, referring to methods for the analysis of covariance. As the globaltest, the procedure of [23] does not explicitly model the correlation structure in the gene set when constructing the test statistic as a combination of gene-wise models. However, the correlation structure is still implicitly maintained because of the use of the sample permutation procedure. Furthermore, a refined follow-up model allows for the explicit inclusion of an estimated correlation matrix [24].

#### Hotelling's *T*^2^-test and related methods

Other procedures for gene set enrichment analysis which are also directly based on the gene set information can be derived from the well-known multivariate extension of the *t*-test, the Hotelling's *T*^2^-test. The corresponding test statistic is

$$\begin{array}{c} T^{2}={\left({\bar{x}}_{1}-{\bar{x}}_{2}\right)}^{T}{\left(S\frac{n}{n_{1}\cdot n_{2}}\right)}^{-1}({\bar{x}}_{1}-{\bar{x}}_{2})\\ =t^{T}R^{-1}t, \end{array}$$

where ${\bar{x}}_{1}$ and ${\bar{x}}_{2}$ are the mean vectors of the gene expressions in the set for the two phenotypes, and *n*_1 _and *n*_2 _are the sample sizes in both groups. ***S ***is the pooled empirical covariance matrix of the genes in the set, and ***R ***is the corresponding correlation matrix. With ***t ***we denote the vector containing all local two-sample equal variance *t*-statistics. Note that as a weighted sum of the gene-wise *t*-statistics Hotelling's *T*^2 ^explicitly incorporates the dependence structure among the genes – unlike most other test statistics for gene set enrichment.

Under the null hypothesis that the mean vectors in both groups are equal, $\frac{n-m-1}{(n-2)\cdot m}T^{2}$ follows an *F *distribution with *m *and *n *- *m *- 1 degrees of freedom, where *m *is the gene set size. However, in the analysis of gene expression data it is rarely the case that the number of samples is greater than the number of genes in the set, so the above procedure needs to be modified appropriately.

Kong *et al. *[25] propose to use dimension reduction by means of singular value decomposition. In effect, their approach amounts to using the Moore-Penrose pseudoinverse for inverting the singular empirical covariance matrix in the definition of Hotelling's *T*^2^.

Another Hotelling-like statistic can be found in [19]. These authors substitute the usual *t*-statistic with the SAM statistic [47]

$$d_{j}=\frac{{\bar{x}}_{j}^{1}-{\bar{x}}_{j}^{2}}{s_{j}+s_{0}},$$

where *s*_0 _is a regularizing constant, and assume a diagonal correlation matrix. This leads to the SAM Gene Set (SAM-GS) statistic

$$\text{SAM-GS}=\sum_{g_{j}\in S}d_{j}^{2}.$$

for a gene set *S*. Significance is assessed by permuting sample labels.

A further possibility is to substitute the empirical covariance matrix in the Hotelling *T*^2 ^statistic with a more efficient estimator. This is important if the sample size is small [48].

### Further procedures

Rahnenführer *et al. *[26] present procedures to investigate enrichment whilst taking into account known pathway structures and co-regulations of gene sets. Adewale *et al. *[30] extend the approach of [19] to account for diverse phenotypes including survival and count data. Lewin and Grieve [28] developed special methods considering the connections between gene sets derived from the graph structure of the Gene Ontology [49]. Procedures to infer the enrichment of gene-gene and gene-protein networks have been presented by Nacu *et al. *[29].

Läuter *et al. *[31] describe a procedure for detecting gene set enrichment and the simultaneous selection of suitable gene sets. This utilizes an exact high-dimensional multivariate spherical test [50]. Note that in this procedure the gene sets are not fixed a priori, but are chosen according to the correlation among genes. As gene set statistic a weighted average of univariate beta statistics is employed.

Edelman *et al. *[27] present an approach similar to the GSEA of [8]. A key feature of their method is that it is constructed to measure the enrichment of gene sets in individual samples. This aims at investigating the variation of the activity of functional groups of genes in the population so that the expression profile of relevant gene sets can be used for predicting the phenotype of an individual. As local test statistic the log-likelihood ratio is used.
